# A Rare Case of Multipathogenic Pneumonia in a Patient With Human Immunodeficiency Virus

**DOI:** 10.7759/cureus.9307

**Published:** 2020-07-21

**Authors:** Ahmad Al-Shyoukh, Moustafa Younis, Mohamed Warsame, Ashraf Gohar

**Affiliations:** 1 Internal Medicine, University of Missouri-Kansas City School of Medicine/Saint Luke's Health System, Kansas City, USA; 2 Internal Medicine, University of Missouri-Kansas City School of Medicine, Kansas City, USA; 3 Infectious Disease, Mayo Clinic, Rochester, USA; 4 Pulmonary and Critical Care and Sleep, University of Missouri-Kansas City (Hospital Hills Campus), Kansas City, USA

**Keywords:** human immunodeficiency virus, pneumocystis carinii pneumonia, cytomegalovirus penumonia, opportunistic infection, strongyloides stercoralis

## Abstract

The incidence of acquired immunodeficiency syndrome (AIDS)-related opportunistic infections has declined dramatically following the introduction of potent antiretroviral therapy (ART). However, pulmonary infections remain a significant cause of morbidity and mortality. The spectrum of pulmonary disease that can affect patients with human immunodeficiency virus (HIV) is wide and includes opportunistic infections with many bacterial, fungal, viral, and parasitic organisms. In this case, we present a 65-year-old woman with HIV, non-compliant with ART, who presented with subacute melena, fatigue, dyspnea, and hemoptysis. After extensive evaluation, she was found to have pneumonia caused by four different pathogens: Strongyloides stercoralis, Pneumocystis jirovecii, Cytomegalovirus (CMV), and Pseudomonas aeruginosa. She received trimethoprim-sulfamethoxazole, steroids, and ivermectin. However, her clinical condition did not improve and she passed away.

## Introduction

Acquired immunodeficiency syndrome (AIDS)-related opportunistic infections have declined in incidence after antiretroviral therapy (ART) was introduced. Lung disorders that happen in patients with human immunodeficiency virus (HIV) vary and include a spectrum of multiple opportunistic infections [1]. They may include bacterial, viral, fungal, and parasitic etiologies, or a combination [2]. Bacterial pneumonia remains very common in HIV patients. However, consideration for other opportunistic infections is necessary. Herein, we present a rare case of pneumonia caused by four different pathogens: Strongyloides stercoralis, Pneumocystis jirovecii, Cytomegalovirus (CMV), and Pseudomonas aeruginosa.

## Case presentation

A 65-year-old female patient with past medical history of HIV infection, hypothyroidism, and medication non-adherence presented to the emergency department (ED) with fatigue and dark stools for two days.

On admission, she reported diffuse abdominal pain, generalized weakness, nausea, non-bilious non-bloody vomiting, hemoptysis, shortness of breath, chronic anorexia, and weight loss. She denied fever, chills, chest pain, diarrhea, or hematuria. She has lived in the Midwest for the past 12 years, previously lived in Mexico. She denied any recent travel, sick contacts, tick bites, camping, hiking, or drinking untreated water.

On examination, her vitals were within normal limits, she appeared cachectic, and had right basilar crackles. The patient did not have a rash.

Complete blood count was remarkable for normocytic anemia with a hemoglobin (Hb) of 6.0 g/dL with lymphopenia and normal eosinophil count (Table 1). Her last CD4 count was 19 cells/µL about five months prior to presentation with a viral load of 1,690,000 copies/mL. 

**Table 1:** Laboratory abnormalities CMV, cytomegalovirus; PCR, polymerase chain reaction; BAL, bronchoalveolar lavage

Lab		Result
Sodium		123 mmol/L
Potassium		3.2 mmol/L
Chloride		93 mmol/L
CO_2_		16 mmol/L
Creatinine		0.80 mg/dL
Blood urea nitrogen		14 mg/dL
Lactate dehydrogenase		181 units/L
Procalcitonin		0.11 ng/mL
Hemoglobin		6.0 g/dL
Mean corpuscular volume		93 fL
White blood cell count		7.70 × 10^3^/cm^3^
Lymphocytes %		4.2%
CMV DNA PCR		80,202 IU/mL
BAL Pneumocystis jirovecii PCR		1,882 copies/mL
QuantiFERON Gold		Indeterminate
Syphilis antibody		Negative
Urine histoplasma antigen		Negative
Blood cryptococcal antigen		Negative

**Figure 1:**
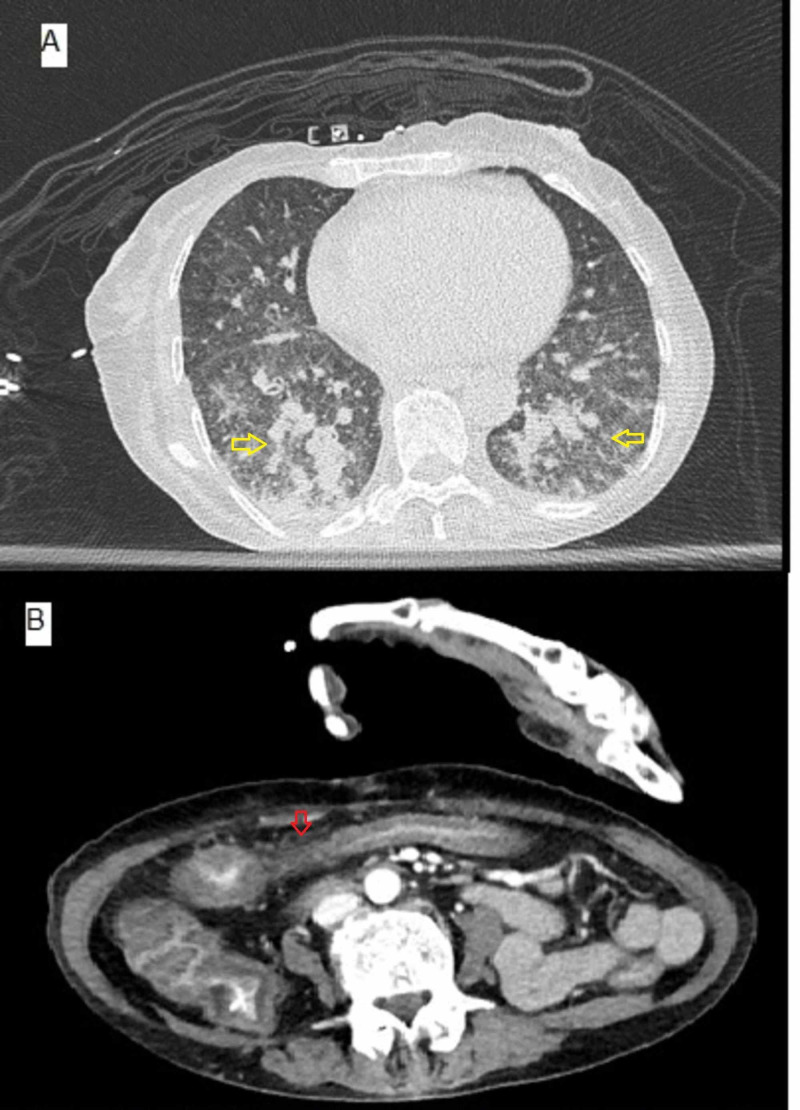
(A) CT chest with bilateral ground-glass opacities (yellow arrows). (B) CT abdomen with colitis (red arrow).

An arterial blood gas was obtained. pH was 7.48, PaCO_2_ was 24.4, PaO_2_ was 73.3 at FiO_2_ of 24%, and HCO_3_ was 17.9. The alveolar-arterial gradient was 67.3 mmHg. CT of her abdomen with contrast and chest without contrast demonstrated severe colitis and diffuse bilateral ground-glass opacities with mildly enlarged mediastinal lymph nodes (Figure 1).

The patient was transfused two units of packed red blood cells and was started on intravenous (IV) levofloxacin and metronidazole for colitis and community-acquired pneumonia. We empirically started Pneumocystis pneumonia (PCP) treatment with trimethoprim-sulfamethoxazole and prednisone due to high clinical suspicion.

Blood cultures including fungal blood cultures were negative. Upper endoscopy and colonoscopy revealed duodenitis and moderate colitis, and biopsies were taken. On hospital day (HD) 3, bronchoscopy was performed with bronchoalveolar lavage (BAL). Subsequently, eosinophilia was noted on HD4 only. On HD 7, BAL was positive for PCP with a PCR of 1,882 quantitative copies/mL. BAL culture grew Pseudomonas aeruginosa, and cytology revealed parasitic larvae consistent with Strongyloides stercoralis and CMV inclusion bodies. The patient was subsequently started on ivermectin. CMV blood polymerase chain reaction (PCR) was positive with 80,202 IU/mL. She was treated with IV ganciclovir. On HD 10, duodenal biopsy also visualized Strongyloides stercoralis. Her respiratory and hemodynamic status steadily declined, and she subsequently developed disseminated intravascular coagulation and septic shock requiring transfer to the intensive care unit. After extensive discussion with the patient’s family, we withdrew care and proceeded with comfort measures. The patient died shortly after.

## Discussion

We present a rare case of multipathogenic pneumonia caused by four different organisms in a woman with HIV. To our knowledge, there were no available reports of patients presenting with four different organisms at once.

The incidence of AIDS-related opportunistic infections has declined dramatically following the introduction of potent ART [1,3,4]. However, pulmonary infections remain a significant cause of morbidity and mortality [1]. Although the spectrum of pulmonary infections is broad and includes opportunistic infection with many bacterial, fungal, viral, and parasitic organisms, bacterial pneumonia remains the most common cause of pneumonia in patients with HIV [1,2]. Prompt appropriate diagnosis and consideration for the possibility of a multipathogenic pneumonia is essential in severely immunosuppressed patients.

Consistent with our patient, patients with HIV with a CD4 count < 50 cells/μL either not receiving or failing ART are at higher risk for CMV infection and/or reactivation [1,5,6]. CMV pneumonitis is uncommon, and its diagnosis is challenging as the signs and symptoms of CMV pneumonia are non-specific [1,2,5,6]. Radiological features include diffuse interstitial or alveolar infiltrates [1,2,6,7]. As a result of viral shedding, it is common for CMV to present in the BAL of patients with HIV; thus, its presence is insufficient for the diagnosis of invasive CMV pneumonitis. The diagnosis is confirmed once BAL cytology or lung biopsy demonstrates cells with inclusion bodies, after other etiologies are excluded [1,2,6]. As such, it is an extremely rare finding and was reported in only 5%-8% of HIV patients undergoing BAL [5]. CMV coinfection has been previously reported in the literature with PCP [8]. CMV pneumonia should be treated similar to other tissue-invasive CMV disease with full treatment doses of antivirals such as IV ganciclovir or IV foscarnet, and the duration of therapy depends on the severity of disease, as well as the clinical and virologic response to treatment ranging from 14 to 28 days or longer [1,6].

CMV pneumonia along with Strongyloides sterocoralis has been reported before in a kidney transplant recipient on immunosuppression [9]. Strongyloides stercoralis is a parasitic nematode that causes gastrointestinal (GI), pulmonary, and disseminated infection in humans. Prevalence is highest in tropical climates and in the southeast part of the US. In a prospective study in the US, 25% of HIV cases were seropositive for Strongyloides stercoralis [10]. Larvae penetrate the skin and are then transmitted through the blood to the lungs where it ascends the tracheobronchial tree and is eventually swallowed entering the GI tract to lay eggs. Eggs hatch into larvae, which are either excreted in the stool or reinfect the same host through the same cycle [1,11]. It mostly manifests as a chronic infection in immunocompetent hosts but can lead to hyperinfection in immunocompromised patients. Hyperinfection is characterized by rapid uncontrolled multiplication of Stronglyoides, resulting in higher disease burden where patients acutely develop severe GI or pulmonary manifestations. Radiologically, bilateral pulmonary interstitial infiltrates are seen. Although eosinophilia is a common finding in chronic infection, it is usually absent in the setting of hyperinfection syndrome [1,12]. Interestingly, peripheral eosinophilia was initially absent in our patient and showed on HD4 following manipulation through bronchoscopy. Hyperinfection syndrome is promoted by the use of corticosteroids as we note in our patient [11]. Her respiratory status decompensated within few days of admission suggesting that steroids initiated on admission might have contributed. The diagnosis of chronic strongyloidiasis is challenging due to the low sensitivity of serology and stool microscopy. In contrast, hyperinfection syndrome is easier to diagnose as large number of larvae are seen in bodily fluids. Management includes supportive care along with anthelmintic medication such as ivermectin or albendazole for at least two weeks [1,11]. Ivermectin results in more cure and is well tolerated [13]. Furthermore, it is important to initiate empiric antibiotics against enteric gram-negative bacteria as Strongyloides may facilitate entry of enteric organisms into the systemic circulation.

Pseudomonas aeruginosa is a common bacterial etiology in pneumonia particularly in patients with CD4 <100 cells/µL [2]. Facilitated by Strongyloides, we suggest that Pseudomonas aeruginosa disseminated from the GI tract to the lungs causing a superimposed pneumonia. Although our patients’ blood cultures were negative, transient bacteremia is a possibility.

PCP is the most common AIDS-defining illness in the US. Its incidence is declining with increased use of ART and PCP prophylaxis but it continues to infect patients unaware of their HIV status or those not adhering to ART or prophylaxis [2,4]. More than 90% of cases in adults occur in those with CD4 count <200 cells/µL [2]. Most patients have an elevated lactate dehydrogenase (LDH), but it can also be normal such as in our patient. It also presents with bilateral interstitial infiltrates on chest imaging, as in our patient; however, diagnosis requires visualization of cysts on either induced sputum or BAL samples [2,14]. The first-line treatment for all severities is trimethoprim-sulfamethoxazole for 21 days. Alternatives for moderate to severe disease include IV pentamidine, clindamycin, and primaquine, and for mild disease include trimethoprim with dapsone, clindamycin with primaquine, or atovaquone. Corticosteroids are also recommended for patients with moderate to severe disease, with PaO_2_ below 70 mmHg on room air, and/or alveolar-arterial gradient above 35 mmHg [14].

## Conclusions

We describe a rare case of HIV-related pneumonia caused by four different pathogens. Multipathogenic pneumonia in severely immunocompromised patients presents a diagnostic and management dilemma and high degree of clinical suspicion is required. Careful clinical evaluation, early diagnosis, and management of pneumonia in HIV patients are pivotal and time sensitive. Considering multiple etiologies in immunocompromised patients is necessary and early bronchoscopy is recommended.
